# Candidate Genes and Gene Networks Change with Age in Japanese Black Cattle by Blood Transcriptome Analysis

**DOI:** 10.3390/genes14020504

**Published:** 2023-02-16

**Authors:** Chencheng Chang, Yanda Yang, Le Zhou, Batu Baiyin, Zaixia Liu, Lili Guo, Fengying Ma, Jie Wang, Yuan Chai, Caixia Shi, Wenguang Zhang

**Affiliations:** 1 College of Animal Science, Inner Mongolia Agricultural University, Hohhot 010018, China; 2 College of Agronomy Animal Husbandry and Bioengineering, Xing’an Vocational and Technical College, Ulanhot 137400, China; 3 College of Life Science, Inner Mongolia Agricultural University, Hohhot 010018, China; 4 Inner Mongolia Engineering Research Center of Genomic Big Data for Agriculture, Hohhot 010018, China

**Keywords:** Japanese black cattle, blood transcriptome, growth, co-expression network, aging

## Abstract

Age is an important physiological factor that affects the metabolism and immune function of beef cattle. While there have been many studies using the blood transcriptome to study the effects of age on gene expression, few have been reported on beef cattle. To this end, we used the blood transcriptomes of Japanese black cattle at different ages as the study subjects and screened 1055, 345, and 1058 differential expressed genes (DEGs) in the calf vs. adult, adult vs. old, and calf vs. old comparison groups, respectively. The weighted co-expression network consisted of 1731 genes. Finally, blue, brown, and yellow age-specific modules were obtained, in which genes were enriched in signaling pathways related to growth and development and immune metabolic dysfunction, respectively. Protein-protein interaction (PPI) analysis showed gene interactions in each specific module, and 20 of the highest connectivity genes were chosen as potential hub genes. Finally, we identified 495, 244, and 1007 genes by exon-wide selection signature (EWSS) analysis of different comparison groups. Combining the results of hub genes, we found that VWF, PARVB, PRKCA, and TGFB1I1 could be used as candidate genes for growth and development stages of beef cattle. CORO2B and SDK1 could be used as candidate marker genes associated with aging. In conclusion, by comparing the blood transcriptome of calves, adult cattle, and old cattle, the candidate genes related to immunity and metabolism affected by age were identified, and the gene co-expression network of different age stages was constructed. It provides a data basis for exploring the growth, development, and aging of beef cattle.

## 1. Introduction

Livestock products are an important source of human food. Beef, as a high-quality dietary protein, has been a high concern for people. With the increase in people’s demand for high-quality beef, researchers have conducted more comprehensive and in-depth studies on cattle [1,2]. Especially with the continuous development of omics, considerable achievements have been made in revealing the genetic characteristics [3] and molecular regulation [4] of beef cattle through whole genome and transcriptome technology. Japanese black cattle have long been known as the representative breed for producing quintessential marbled beef [5]. Previous studies have focused on improving the yield of quality marble beef [6], mechanisms of intramuscular fat deposition [7], genetic trait improvement [8], and feeding management strategies [9]; however, studies on metabolism and immunity were rarely reported. A recent study used transcriptome technology to analyze gene expression and biological function in different tissues and specific developmental stages of Japanese black cattle, revealing the molecular mechanism of important economic traits [10]; however, blood, as an important tissue involved in growth, metabolism, and immune regulation [11], was not included in this study.

As we all know, blood is involved in various physiological processes and plays a vital role in the life cycle of animals [12]. According to previous studies, blood cells express about 80% of the genes found in vital tissues such as the brain, heart, and liver [13]; therefore, blood is increasingly being used as a vehicle for the development of molecular markers associated with traits or regulation [14]. The identification of molecular markers in bovine blood by transcriptome has been reported in recent years, including studies on genes associated with milk yield in Holstein cows [15], candidate genes of short distance transported a stress response in Qinchuan cattle [16], and molecular markers of postpartum disease in different parities of Japanese black cows [17]. However, the effect of age-associated gene changes in blood has only been reported in humans [18], pandas [19], and African green monkeys [20]. With aging, far-reaching changes occur in the animals’ metabolism and immune system, which researchers call “senescence”. Genes in the blood also showed different expression levels at different ages [21]. Therefore, studies on blood transcriptome in beef cattle at different ages is helpful in understanding how immunity and metabolism vary with age, as well as the molecular mechanisms underlying these changes.

The objective of this study was to identify candidate genes and regulatory networks related to metabolism, and immunity affected by age, by comparing the blood transcriptome of calf, adult, and aged cattle, and to explore molecular markers for the growth and aging process of beef cattle. We expected to provide a fundamental basis for Japanese black cow breeding and also to provide a data reference for perfecting marker-assisted management strategies for breeding and enhancing the feeding initiative of pastures.

## 2. Materials and Methods

### 2.1. Laboratory Animal and Sample Collection

A total of 45 Japanese black cows from three age stages (calf, adult, and old) were used in this study, including six calves at 60 days old, 26 adult individuals (3 years old), and 13 old cattle (9 years old). All cattle were raised in Yuan Niu Reproductive and Breeding Technologies Co., Ltd (Hohhot, Inner Mongolia, China). under the same feeding situations and conditions. Blood was collected from the tail vein of all experimental cattle, and all samples were immediately frozen with liquid nitrogen for total RNA extraction.

### 2.2. RNA Extraction, Sequencing, and Data Analysis

Total RNA was extracted from all samples by using TRIzol Reagent following the manufacturer’s instructions. After the RNA samples passed the quality inspection, the Illumina mRNA-seq library kit was used for continuous specific transcriptome library construction, then the double-stranded cDNA was purified, and the quality of the cDNA library was detected by Agilgennt 2100. The library that met the sequencing standard was paired-end sequenced using the Illumina HiSeq 2000 platform. The quality of raw data was evaluated using the FastQC program. High-quality clean reads were obtained after removing raw reads with more than 5% unknown nucleotides and other low-quality reads with even lower quality scores. Subsequently, the clean data were mapped to the reference genome (*Bos taurus* ARS-UCD1.2) by the HISAT2 v2.2.1 [22]. The genome localization information of the bovine reference genome was used to calculate the alignment between effective reads and gene regions. SAMtools v1.9 was used to sort BAM-aligned files generated from HISAT2 by name [23]. StrigTie v2.1.1 was used to calculate the read count for each sample and normalize the reads to FPKM (fragments per kilobase of exon model per million mapped fragments) [24].

### 2.3. Differentially Expressed Genes (DEGs) Analysis

DEGs across three age stages (calf vs. adult, adult vs. old, calf vs. old) were detected using DESeq2 v1.30.1 in the R package with default parameters [25]. The genes complied with|log_2_FC| > 1 and *p* < 0.05 standard were screened out as DEGs. DEGs from three comparisons were pooled, and redundant duplicate genes were removed for subsequent analyses. The correlation between the expression of DEGs and the sample was calculated using BioLadder (https://www.bioladder.cn accessed on 20 June 2022), and the heatmap was used as a visualization of the results.

### 2.4. Weighted Gene Co-Expression Network Analysis

WGCNA (weighted gene co-expression network analysis) is an analytical method for analyzing gene expression patterns in multiple samples that can cluster genes with similar expression patterns and analyze associations between modules and specific traits or phenotypes [26]. In this study, we used genes in the differential list for co-expression network analysis by the TBtools R package [27]. When the fit index was 0.85, an appropriate value for the scale-free network construction was determined. A dynamic tree-cutting algorithm for module division was used, with a minimum number of genes in each module of at least 50 and a threshold of 0.25 for similar module merging. Furthermore, we identified stage-specific modules with strong correlations between GS and MM values (*p*-value < 0.05) and highly correlated module-trait relationships (correlation coefficient > 0.5).

### 2.5. Functional Enrichment and PPI Analysis

For genes in specific modules, the Kyoto Encyclopedia of Genes and Genomes (KEGG) pathway and Gene Ontology (GO) analyses were conducted using KOBAS (https://kobas.cbi.pku.edu.cn accessed on 30 June 2022) and DAVID (https://David.ncifcrf.gov. accessed on 30 June 2022). GO terms and pathways with a *p*-value < 0.05 were defined as significantly enriched. Genes were calculated by STRING, and the protein-protein interaction (PPI) network was obtained and imported into Cytoscape. Node sizes and colors indicated different node degrees, and the width of the edges indicated combined scores and intra-module connection weights.

### 2.6. Exon-Wide Selection Signature

Transcriptome-level variant analysis can help us locate relevant potential functional genes. Although the number of coding regions SNPs were small, any base change in the exon may affect the translation and phenotypic traits of the protein. Therefore, SNP had profound significance in the study of trait expression and phenotypic variation. The exon-wide selection signature (EWSS) was an efficient mutation detection method for SNP detection and screening based on the transcription level, using the fixation index (Fst) as an indicator to measure the degree of population differentiation [28]. The population differentiation index was calculated by the Vcftools program, keeping the checkpoint with positive Fst to determine the SNP selection signal of different groups (Fst > 0.15). The sliding window method was used to locate candidate genes.

## 3. Results

### 3.1. Data Analysis of Transcriptome

A total of 286.9 Gb raw reads in 45 samples from three age stages by RNA-seq, 250 Gb clean data were obtained through quality control. The mapping rate was approximately 92.87% (ranging from 87.23% to 94.88%) after aligning clean reads to the reference genome (ARS-UCD1.2) (Supplementary Table S1). We acquired 22,145 genes for further analysis after deleting the data that was not expressed in all the samples in Supplementary Table S2. Supplementary Figure S1 showed the gene expression distribution of three age stages.

### 3.2. Differentially Expressed Genes across Three Age Stages

**Figure 1:**
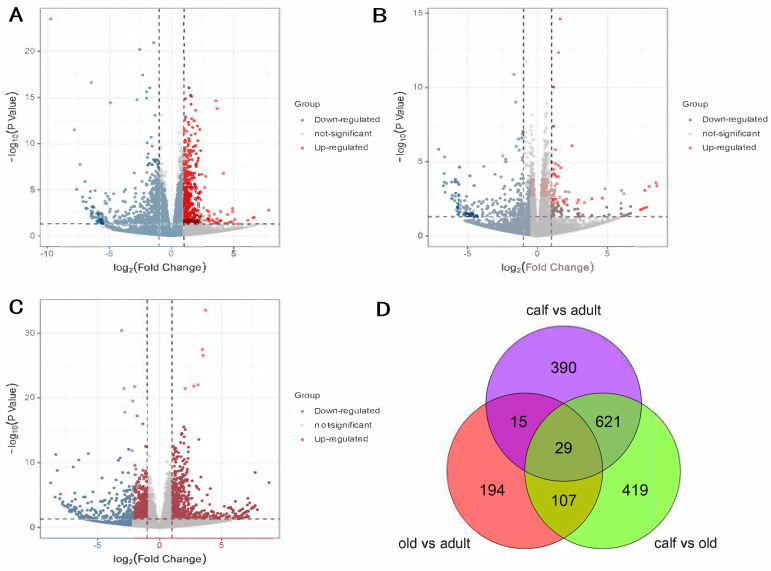
(**A**) volcano plot for DEGs in calf vs. adult. (**B**) volcano plot for DEGs in adult vs. old. (**C**) volcano plot for DEGs in calf vs. old. (**D**) Venn diagram of DEGs in each group.

Differential expression genes were identified by performing a pairwise comparison among the three groups. In the calf vs. adult comparison, 1055 DEGs in total, comprising 450 up-regulated genes and 605 down-regulated genes were observed (Supplementary Table S3 and Figure 1A). In the comparison of adults and old, 345 DEGs were found, that with the least DEGs among the three comparisons, including 228 down-regulated genes and 117 up-regulated genes (Supplementary Table S4 and Figure 1B). The comparison between calf and old showed the highest amount of DEGs, where 1158 DEGs were observed, containing 515 up-regulated genes and 643 down-regulated genes (Supplementary Table S5 and Figure 1C). A total of 29 DEGs (Figure 1D) were shared for three comparisons, and 1731 genes were retained for subsequent WGCNA analysis after removing duplicates. The hierarchical clustering heatmap of 1731 DEGs was presented in Supplementary Figure S2, showing that calf had a low correlation with adult and old, while adult showed a strong correlation with old.

### 3.3. Construction of Weighted Gene Co-Expression Network and Module Detection

**Figure 2:**
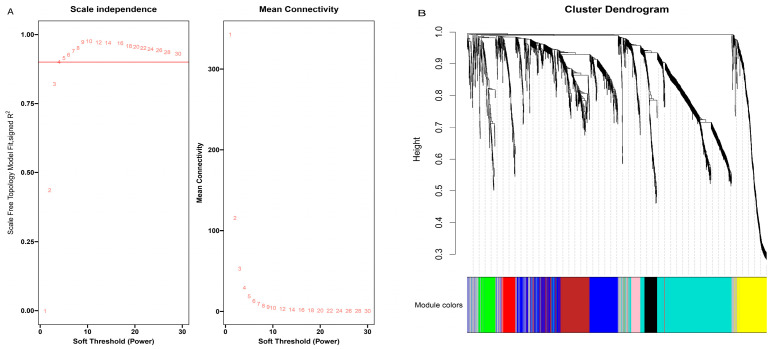
Scale independence and mean connectivity of co-expression network. (**A**) Screening of soft thresholds. (**B**) Cluster dendrogram.

The relationship and function of DEGs in the three age groups can be properly appreciated using WGCNA analysis. The co-expression analysis in this study used 1731 DEGs, according to scale independence and mean connectivity measurements, and confirmed that the soft threshold (β) = 4 and the scale-free network fitting index (R^2^) was greater than 0.85 (Figure 2A), corresponding to the characteristics of the scale-free network. The block-wise module function was used to allocate 1731 differential genes to 9 modules (Figure 2B), and the number of genes in each module varied substantially, from 52 genes in the pink module to 483 in the turquoise module.

### 3.4. Identification of Specific Modules for Each Age Stage

**Figure 3:**
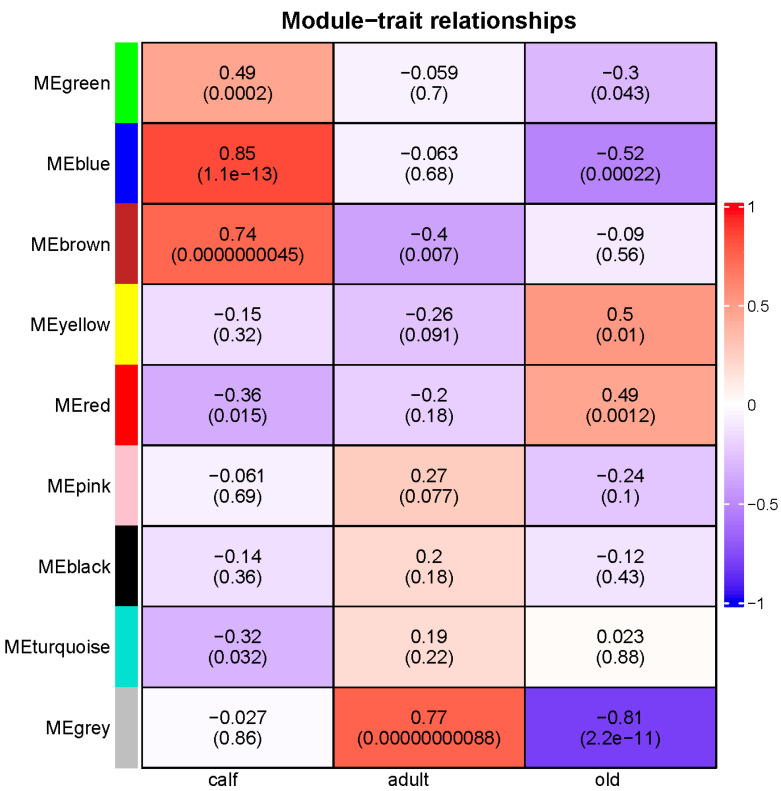
The correlation between the differentiation period and the modules. Red is positive and blue is negative.

By calculating GS and MM, two crucial metrics in the co-expression network analysis, the correlation between them determines whether the genes associated to a trait play a significant role in the stage-specific module. As a result of using |R^2^| > 0.5 and *p* < 0.05 as the screening condition, three stage-specific modules were obtained, namely the blue module, yellow module, and brown module were shown in Figure 3. The brown module was positively correlated with calf stage (R^2^ = 0.74), and the yellow module was negatively correlated with the old stage (R^2^ = 0.50). As a specific module of both the calf stage and the old stage at the same time, the blue module had a significant positive correlation with the calf (R^2^ = 0.85), but a significant negative correlation with the old (R^2^ = −0.52). However, none of the modules had a correlation greater than 0.5 with the adult stage.

### 3.5. Functional Enrichment of Stage-Specific Modules

**Figure 4:**
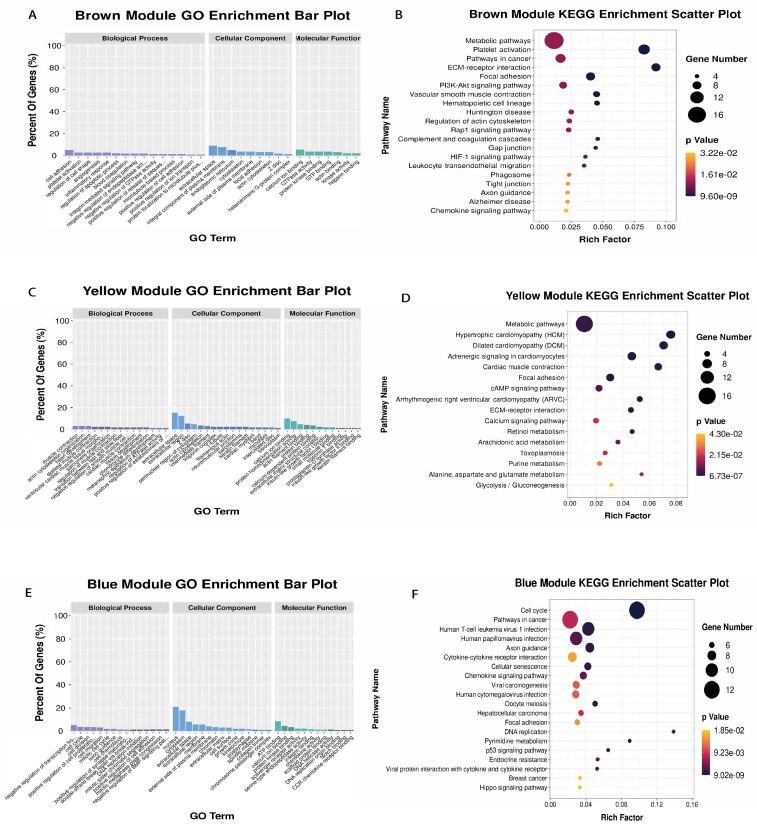
Results of GO analysis and KEGG enrichment in related specific module genes. (**A**) GO enrichment results of genes in brown module. (**B**) KEGG results of genes in brown module. (**C**) GO enrichment results of genes in yellow module. (**D**) KEGG results of genes in yellow module. (**E**) GO enrichment results of genes in blue module. (**F**) KEGG results of genes in blue module.

**Figure 5:**
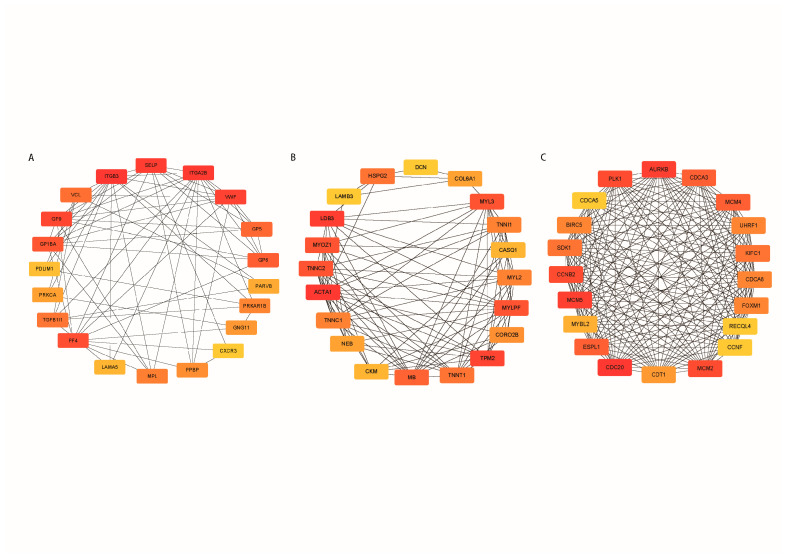
Visualization of potential genes in stage-specific modules. (**A**) PPI network for brown module. (**B**) PPI network for yellow module. (**C**) PPI network for blue module. Different colors indicate the level of genetic connectivity, with red indicating high connectivity and yellow indicating low connectivity.

GO and KEGG pathway enrichment analysis was carried out on the modules related to various ages in order to better understand the biological functions of each module. The brown module is specific for the calf stage. The enrichment results of this module mainly focused on GO terms and pathways related to growth and metabolism, including metabolic pathways, platelet activation, PI3K-Akt signal pathway, ECM-receptor interaction, and hematopoietic cell lineage (Figure 4A,D), suggesting that genes involved in cell proliferation and metabolism form a co-expression network and jointly regulate the growth process during the growth period of calves. The yellow module is specific for the old stage. Metabolic pathways, hypertrophic cardiomyopathy (HCM), dilated cardiomyopathy (DCM), retinol metabolism, arachidonic acid metabolism, and the glycolysis/gluconeogenesis pathway were mainly enriched, indicating that heart function decreased and nutrient metabolism decreased during the aging stage of cattle (Figure 4B,E). Gene enrichment results of the blue module, which was positively correlated with calf stage and negatively correlated with old stage, included both growth- and senescence-related aspects, including cell cycle, DNA replication, cellular senescence, and the NF-kappa B signaling pathway (Figure 4C,F). Detailed GO and KEGG results can be found in Supplementary Tables S6 and S7. The PPI interactions between genes within the module were visualized using the CytoHubba plugin in cytoscape, and the top 20 genes with the strongest connectivity were selected as candidate hub genes (Figure 5A–C).

### 3.6. Exon-Wide Selection Signature

**Figure 6:**
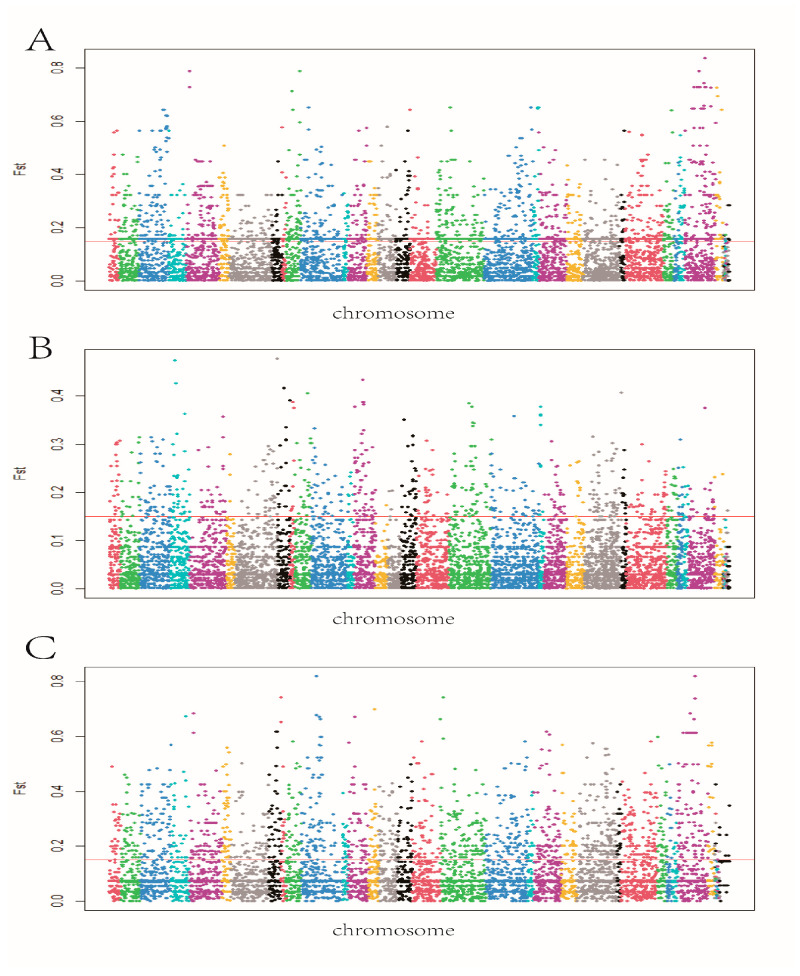
(**A**) Manhattan plots of SNPs in calf compared with adult. (**B**) Manhattan plots of SNPs in adult compared with old. (**C**) Manhattan plots of SNPs in calf compared with old.

SAMtools and BCFtools software were used in this study to check for SNP in cattle of various ages. In order to compare the variations in genetic variation and allele frequencies among various populations, the population differentiation index (Fst) was used as the filtering criterion, and the SNPs with high differentiation degrees were annotated. Manhattan plots (Figure 6) showed the results of the exon-wide selection signature, with highly divergent SNPs defined above the horizontal line (Fst > 0.15). The resulting SNPs were aligned to the reference genome (ARS-UCD 1.2), and a sliding window of 1000 kb was used for gene annotation; 495, 244, and 1007 selected genes were obtained for each of the three comparison groups (Supplementary Table S8).

### 3.7. Candidate Genes for Each Age Stage

**Figure 7:**
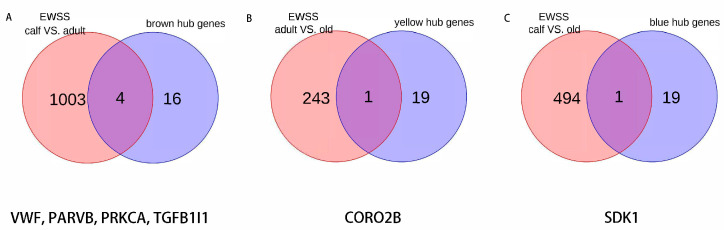
(**A**) Venn diagram of EWSS calf vs. adult and brown module hub genes. (**B**) Venn diagram of EWSS adult vs. old and yellow module hub genes. (**C**) Venn diagram of EWSS calf vs. old and blue module hub genes.

According to the enrichment results of specific modules in our previous step, the signaling pathways related to growth and aging were significantly enriched, indicating that the specificity of the modules was strong. Combined with the results of the EWSS between the comparison groups, we have reason to think that genes with both the hub gene list and EWSS mutation results can be used as candidate genes for different age stages, or growth and aging stages (Figure 7). *VWF*, *PARVB*, *PRKCA*, *TGFB1I1* and *CORO2B*, *SDK1* corresponds to candidate genes in the growth and aging stages, respectively.

## 4. Discussion

In order to determine candidate genes for growth and aging stages and investigate the regulatory mechanisms associated to age changes in Japanese black cattle, we utilized 45 blood samples from Japanese black cattle at different ages to establish transcriptional profiles in this research. For the purpose of constructing a co-expression network, we used a variety of analysis methods to identify the genes that were expressed differently in different ages of Japanese black cattle. Signal pathways related to growth and aging were significantly enriched through enrichment analysis of the specific modules of the co-expression network. We also used exon-wide selection signature analysis to further narrow down the range of candidate genes, and finally obtained four candidate genes (*VWF*, *PARVB*, *PRKCA*, *TGFB1I1*) related to growth, and two candidate genes (*CORO2B*, *SDK1*) which can be used as aging markers.

The physiological health of livestock in the growth and development stage is the biological basis for its ability to produce high-quality products [29,30]. The changes of biological characteristics and hematological parameters that play a major role in normal development are age-related [31], and these changes to a certain extent determine the service life of livestock. Blood is a complex liquid connective tissue synthesized from various cells in the body [32] and can be used as a driving force for the organism to regulate its functions. Because blood samples are readily available and non-invasive, researchers use whole blood to study relevant molecular biomarkers [13] to evaluate aspects of animal function at any age, in any functional state, and under any environmental conditions [33,34]. The blood transcriptome reflects expression profiles associated with physiological changes in other tissues based on gene expression in whole blood [35]. Previous studies have used blood samples for extensive analyses, revealing the relationship between age and gene expression and exploring the mechanism of growth and aging [36]. Similar to this study, most are based on several age nodes to explore the specificity of genes expressed at different periods. What is different is that few studies have used EWSS to explain these issues from the perspective of single nucleotide polymorphisms. The advantage of this approach is that it does not simply rely on the gene expression to be quantified, but instead focuses on the variation in the exon region, which can better explain the causal relationship [37,38].

Signaling pathways are a series of enzymatic reaction pathways that can transmit extracellular molecular signals through the cell membrane to the cell to exert effects and can comprehensively reflect the biological processes that cells are experiencing [39]. Our results showed that growth and development related pathways were significantly enriched during the process from calf to adult, especially the PI3K-AKT pathway. It is an intracellular signal transduction pathway that promotes metabolism, proliferation, cell survival, growth, and angiogenesis in response to extracellular signals. It is a process mediated by serine or threonine phosphorylation of a range of downstream substrates [40]. A considerable number of studies have shown that the PI3K-AKT is a key pathway regulating bovine somatic cell proliferation. Diniz et al. studied the effects of different nutritional levels in pregnant cows on fetal development and found that the PI3K-AKT pathway was negatively affected when maternal nutrition was insufficient, thus inhibiting fetal growth [41]. Along with the ECM-receptor interaction pathway, the PI3K-AKT pathway regulates adipocyte proliferation in beef cattle and takes part in the process of fat deposition [42]. The ECM-receptor interaction pathway that interacts between the ECM and cell surface receptors regulates cell behavior and plays an important role in intercellular communication, cell proliferation, adhesion, and migration [43]; it was also one of the pathways enriched at the growth stage in this study. ECM-receptor interaction has frequently been connected to signaling pathways that are closely related to meat quality in studies of beef cattle [44]. In addition, ECM-receptor interaction is involved in the regulation of muscle growth during the early growth stage of broilers [45] and also plays a key role in calf growth [46]. Aging is a general term for the decline of immune metabolism in animals and is not often mentioned in cattle studies. A typical aging-related signaling pathway is NF-κB, an ancient host defense system involved in immune response and response to multiple external and internal danger signals, such as oxidative stress, hypoxia, and genotoxic stress [47]. Many studies have shown that aging in mammals is associated with activation of the NF-κB transcription factor system [48]. Immunosenescence is a typical age-related decline in the function of the immune system. The NF-κB system plays an important role in regulating both innate and adaptive immunity, such as NF-κB signaling is involved in T cell development, activation, and proliferation. Interestingly, some longevity-related genes inhibit NF-κB signaling, which can delay the aging process and prolong the life span [49].

Growth traits have always been important traits in the breeding and improvement of beef cattle. With the deepening of research on beef cattle, researchers have also conducted more detailed research on the growth of beef cattle. In this study, we conducted a comprehensive analysis of the growth and development process of Japanese black cattle, and identified candidate genes related to calf growth. Some genes are similar to the previous research results and directly participate in the growth and development process, while others indirectly affect the co-expression network. *TGFB1I1*, Transforming Growth Factor β 1 Induced Transcript 1, a multifunctional cytokine that regulates cell proliferation, differentiation, and production of the extracellular matrix, has an effect on development, wound healing, organ fibrosis, tumor generation, and metastasis [50]. In different types of cells, TGF-β1 may have different effects, inhibiting the proliferation of epithelial cells while promoting the proliferation of mesenchymal derived cells [51]. As a key gene regulating growth and development, *TGFB1I1* has also been frequently mapped in ruminant animals. Studies have shown that *TGFB1I1* is involved in bovine ovarian development [52], and it is also known to be a key regulator of liver inflammatory response and muscle tissue development [53]. *TGFB1I1* played an important role in the high-feed efficiency group, and similar results were obtained in the buffalo study [54]. *PRKCA* is a family of serine- and threonine-specific protein kinases that can be activated by calcium and the second messenger diacylglycerol [55]. Each member of the *PKC* family has a specific expression profile and is believed to play a distinct role in cells [56]. This kinase has been reported to play roles in many different cellular processes, such as cell adhesion, cell transformation, cell cycle checkpoint, and cell volume control [57]. In the study of intramuscular fat deposition in yaks, *PRKCA* has been used as a target gene involved in the process of fat deposition [58], and it is also an important candidate gene for growth rate of broilers [59]. *PARVB* encodes a member of the parvin family of actin-binding proteins, which play a role in cytoskeleton organization and cell adhesion [60]. Overexpression of *PARVB* was found to increase cell migration ability [61].

Aging refers to a progressive multifactorial decline in function over time at the molecular, cellular, tissue, and organism levels [62]. The aging organism becomes weak and its susceptibility to disease increases [63]. Aging is a major risk factor for aging-related diseases, including neurodegeneration, cardiovascular disease, osteoporosis, and cancer [64]. This process depends on the interplay between numerous genetic, environmental, and lifestyle factors. The molecular mechanisms of aging can be attributed to cumulative genetic mutations and epigenetic dysfunction [65]. These molecular alterations directly interact with the transcriptional network. Therefore, it is important to identify molecular features associated with aging to improve our understanding of aging and its associated diseases. *CORO2B*, which we identified during bovine aging, is a podocyte protein involved in the regulation of the actin cytoskeleton, mainly in the kidney, and indirectly involved in the regulation of carbohydrate metabolism [66]. The sidekick cell adhesion molecule 1 (*SDK1*) gene belongs to the immunoglobulin superfamily (IgSF) and is defined as a disease-associated missense mutation in the genome of Chinese local cattle [67].

## 5. Conclusions

In this study, we performed transcriptome analysis using 45 Japanese black cattle, which were divided into calf, adult, and aged groups according to age. By comparing the differentially expressed genes between the groups, it was found that there were large biological differences in different age stages. We then performed WGCNA analysis on the differentially expressed genes, constructed gene co-expression networks in different age stages, and identified hub genes in growth and development and aging stages. Finally, from the genomic perspective, EWSS analysis was used to identify the variation information between different groups. By integrating the results of WGCNA and EWSS analyses, we identified *VWF*, *PARVB*, *PRKCA*, and *TGFB1I1* as candidate genes for the growth and development stage of beef cattle, and *CORO2B* and *SDK1* as candidate genes for the decline of immuno-metabolic function and the aging process of beef cattle. These results are worthy of further verification and provide a theoretical basis for breeding Japanese black cattle.

## Data Availability

All data are presented in the article, and the original data can be obtained by email asking the author.

## Supplementary Materials

The following Supporting Information can be downloaded at: https://www.mdpi.com/article/10.3390/genes14020504/s1, Figure S1: Diagram of gene expression distribution; Figure S2: Hierarchical clustering heatmap of DEGs; Table S1: Number of reads, and number of aligned reads, per cattle sample; Table S2: Gene expression matrix; Table S3: Number of differentially expressed genes between calf and adult; Table S4: Number of differentially expressed genes between adult and old; Table S5: Number of differentially expressed genes between calf and old; Table S6: GO enrichment for specific modules; Table S7: KEGG enrichment for specific modules; Table S8: Number of EWSS mapped genes in different periods.
