# Supplementary figures and images for: Candidate Genes and Gene Networks Change with Age in Japanese Black Cattle by Blood Transcriptome Analysis
Source: Genes (Basel). 2023 Feb 16;14(2):504. doi: 10.3390/genes14020504 (PMC9956108; doi:10.3390/genes14020504)

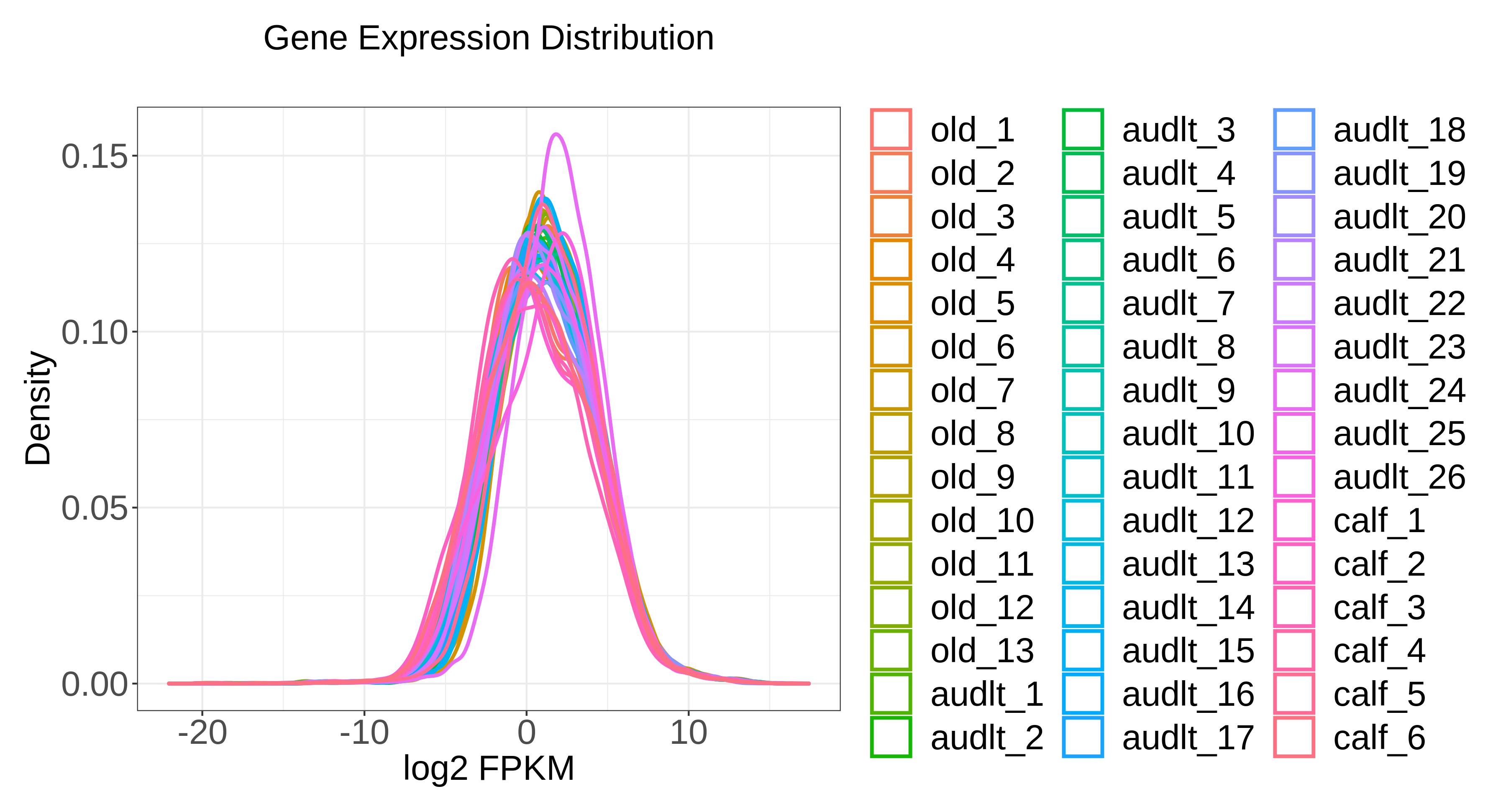

Supplement: Supplementary file 1 [file genes-14-00504-s001.zip › Supplementary Figure S1.jpeg]

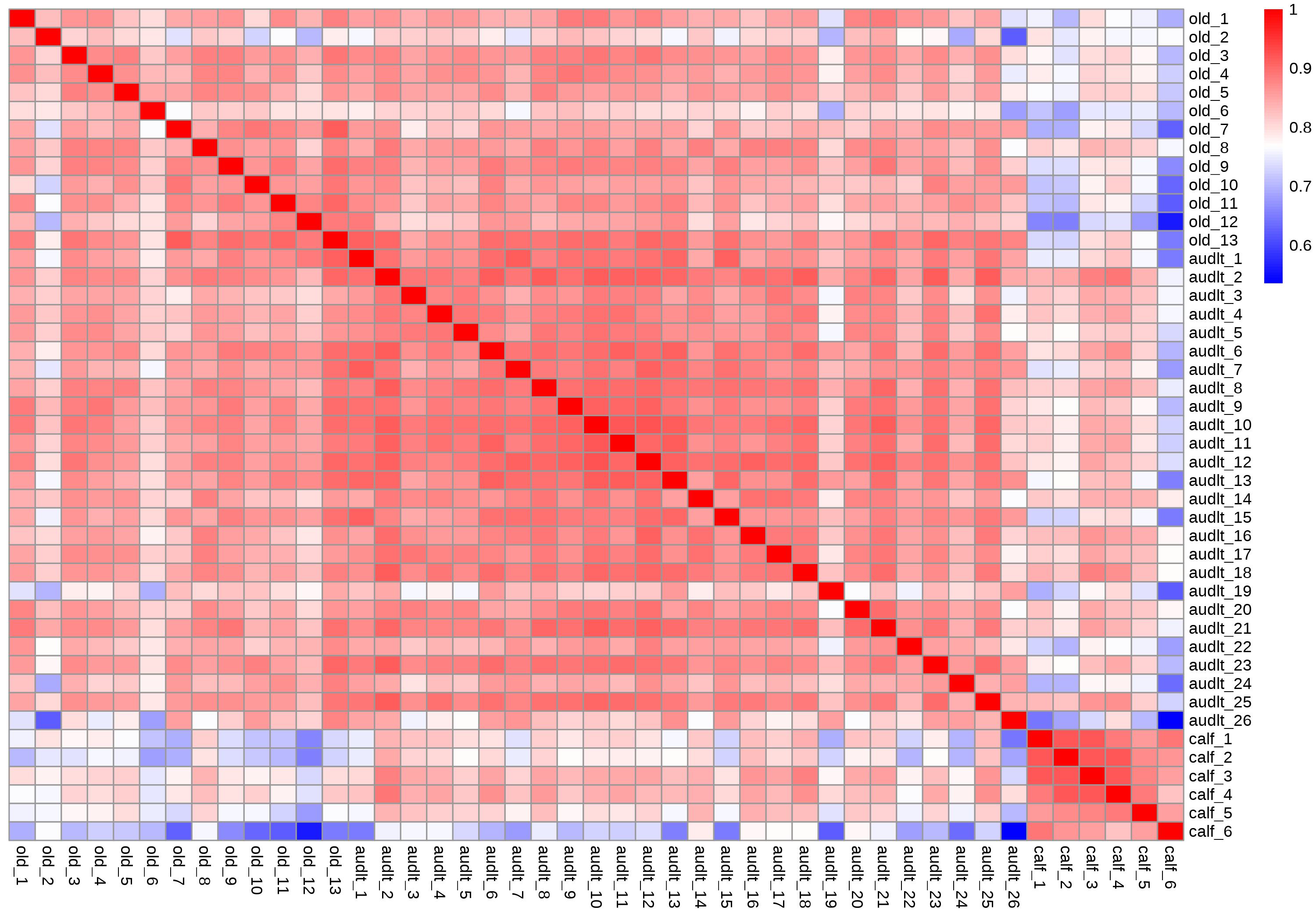

Supplement: Supplementary file 1 [file genes-14-00504-s001.zip › Supplementary Figure S2.jpg]
